# Conversion of Biological Waste into Porous Carbon with Hierarchical Porous Architecture for High-Performance Supercapacitors

**DOI:** 10.3390/nano16161035

**Published:** 2026-08-20

**Authors:** Yueyang Lu, Siyu Han, Yizhe Wang, Zekun Tang, Xiaoliang Wu

**Affiliations:** 1 State Key Laboratory of Utilization of Woody Oil Resource, College of Chemistry, Chemical and Resource Utilization, Northeast Forestry University, Harbin 150000, China; 15736877356@163.com (Y.L.); wangyizhe05@vip.163.com (Y.W.); tangzekun2004@163.com (Z.T.); 2 Jiamusi Ecological Environment Technology Center, No. 676 Chang’an West Road, Jiamusi 150040, China

**Keywords:** reed panicle, KHCO_3_, ammonium borate, supercapacitor

## Abstract

Biowaste-derived porous carbon materials show promise as electrode materials for supercapacitors owing to their low cost, renewable nature, widespread availability, and high specific surface area. Herein, boron and nitrogen co-doped porous carbon was synthesized via a facile one-step activation approach using reed spike as carbon precursor, ammonium borate as both the nitrogen and boron source, and potassium bicarbonate as activator. The prepared PC-700 materials possess large specific surface areas with hierarchical porous architectures and rich N (2.54 at%), O (11.23 at%) and B (2.59 at%) functional groups. As an electrode material, the PC-700 materials show a specific capacitance of 329.6 F g^−1^ at 0.5 A g^−1^ and long lifespan. Notably, the assembled PC-700 symmetric super capacitor achieves an energy density of 20.5 Wh kg^−1^ and excellent electrochemical stabilization (98.60% capacity retention after 10,000 cycles).

## 1. Introduction

Global energy demands continue to rise, while over-reliance on fossil fuels (such as coal, oil, and natural gas) has not only led to resource depletion and price volatility but also exacerbated environmental crises like global warming and extreme weather, posing severe threats to biodiversity and ecosystem stability [1,2]. To address these challenges, the world is actively transitioning to renewable energy sources such as solar, wind energy, etc. However, the intermittent and fluctuating nature of these sources, due to their dependence on natural conditions, necessitates the use of efficient energy storage devices for energy buffering and stable supply [3]. Among different energy storage devices, super capacitors have drawn significant attention and have experienced rapid development owing to their unique advantages, including high power density, fast charge–discharge capability, and their exceptionally long cycle life [4,5,6,7,8,9]. Activated carbon, as the preferred material for super capacitors, possesses large specific surface area and superior electrochemical stabilization. Nevertheless, activated carbon is usually prepared by first carbonizing and then activating with strong alkali. The pore structure of activated carbon is composed mainly of dendritic and tortuous micropores, with a long diffusion distance and high diffusion resistance, leading to poor electrochemical performance [10,11,12,13].

As is known, the pore structure regulation of carbon materials can significantly improve their electrochemical performance [14,15,16]. Designing a hierarchical porous structure with micropores, mesopores, and macropores can achieve improved electrochemical performance [17,18,19]. Generally, micropores can form interface double layers for energy storage, while mesopores can serve as ion high-speed channels, and macropores can serve as ion buffer pools to shorten the distance of ion diffusion [20,21,22,23]. Furthermore, the preparation of hierarchical porous carbon using biological waste as precursor has received widespread attention in recent years. Shang et al. used houttuynia as precursor to prepare 3D N-doped hierarchically porous carbon materials and the obtained electrode materials show high capacitance [24]. Furthermore, doping carbon materials with heteroatoms can not only improve their hydrophilicity but also provide some pseudocapacitance [25]. In particular, the band gap between nitrogen and carbon atoms is relatively small, which enables N-doped porous carbon to have high electron transfer rates and enhance their conductance. After nitrogen doping into the carbon framework, four kinds of bond states are generated (N-6, N-5, N-Q and N-X). N-Q can enhance the sample’s conductivity, while N-6 and N-5 can provide the pseudocapacitance [26]. Huang et al. prepared nitrogen-doped porous carbon (NPC) by carbonization of the mixture of Auricularia auricula, zinc chloride, and ammonium chloride [27]. When compared with the porous carbon without nitrogen doping, the specific capacitance of NPC increased from 280 F g^−1^ to 347 F g^−1^. Moreover, boron as a p-type dopant can enhance the hydrophilicity and conductivity of porous carbon, as well as provide pseudocapacitance. Peng et al. synthesized B/N co-doped porous carbon through the H_3_BO_3_-mediated activation of N/O-rich polymer. The research results indicate that boron doping can greatly enhance the electrochemical feature [28].

Reeds are a valuable biological waste resource and are commonly found in shallow or low-lying wetlands worldwide, with strong regenerative capacity and vigorous vitality. Under natural conditions, they produce 10–13 tons/hectares of fresh grass. Transforming reed spikes into high-performance porous carbon electrodes not only achieves efficient utilization of biological waste but also reduces environmental pollution. Reed straws were selected as the biomass precursor owing to their natural hollow tubular architecture and abundant lignocellulosic content. The natural hollow channels in reed-derived carbon precursors have been demonstrated to facilitate the formation of interconnected through-pore architectures during activation, which are highly beneficial for electrolyte ion transport [29,30,31]. However, the reed panicles have been rarely investigated as a precursor for advanced energy storage materials. Rather than treating reed as mere waste, our approach recovers value from each component, simultaneously boosting resource efficiency and easing the burden of agricultural residue disposal, all while reinforcing circularity.

This study utilizes reed panicles as the biomass carbon precursor, ammonium tetraborate as nitrogen and boron source, and potassium bicarbonate as the activator. The prepared PC-700 materials show high specific surface area with hierarchical porous architecture and rich N (2.54 at%), O (11.23 at%) and B (2.59 at%) functional groups. The PC-700 electrode shows a specific capacitance of 329.6 F g^−1^ at 0.5 A g^−1^ and has a long lifespan. Notably, the assembled PC-700 symmetric supercapacitor achieves an energy density of 20.5 Wh kg^−1^ and excellent electrochemical stabilization (98.60% capacity retention after 10,000 cycles).

## 2. Experimental Section

### 2.1. Materials

Reed panicle, ammonium borate, potassium bicarbonate, hydrochloric acid (HCl), polytetrafluoroethylene (PTFE), and ethanol were of analytical grade and were obtained from Sinopharm Chemical Reagent, Co., Ltd. (Shanghai, China). All raw materials were used directly as purchased without further purification. The water used in the experiments was freshly deionized.

### 2.2. Preparation of N, B Co-Doped Porous Carbon

**Scheme 1:**
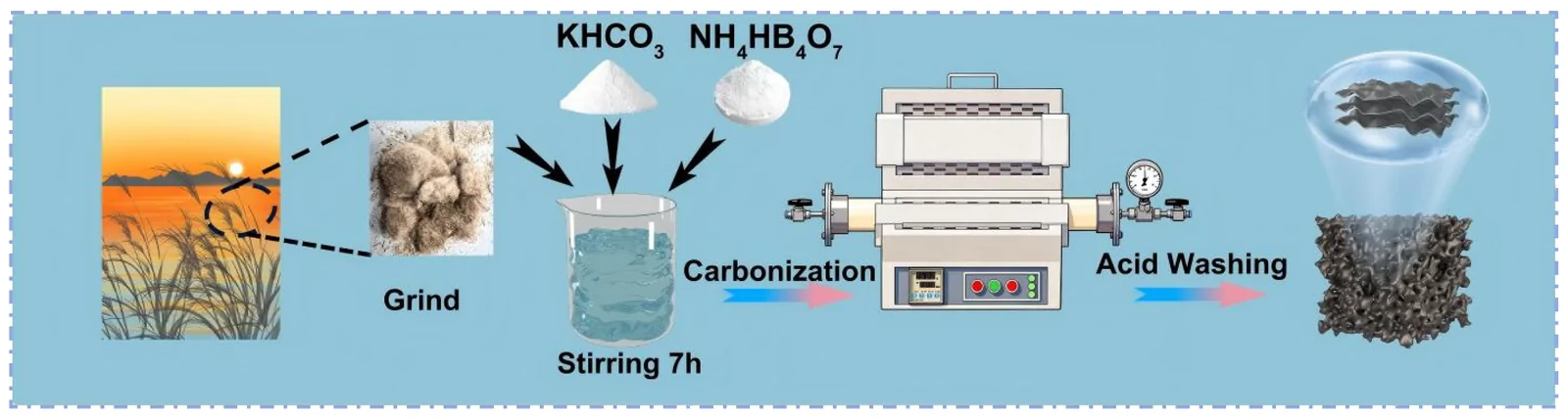
The synthesis process of PC samples.

Scheme 1 demonstrates the synthesis process of N, B co-doped porous carbon. A facile one-step activation approach was conducted that had been proven by other researchers previously. The collected reed panicles were pulverized into powder using a multi-functional grinder and were subsequently stored in sealed preservation bags. Amounts of 2 g of ammonium borate (NH_4_HB_4_O_7_·3H_2_O) and 2 g of potassium bicarbonate (KHCO_3_) were dissolved in 50 mL of deionized water under vigorous stirring. Ammonium borate serves as a dopant and introduces boron and nitrogen heteroatoms into the carbon framework during carbonization. Meanwhile, KHCO_3_ functions as an activator that decomposes during carbonization to release CO and K, creating additional micropores and mesopores through chemical etching. Subsequently, 2 g of reed panicle powder was added to the solution and the mixture was dried at 60 °C for a period. The raw materials were transferred into a high-temperature tubular furnace and heated to 600 °C, 700 °C, and 800 °C for 2 h in nitrogen with a heating rate of 5 °C min^−1^. The samples were washed with diluted HCl and deionized water. Finally, each obtained sample is filtered and dried in an oven. The final products were labeled PC-600, PC-700, and PC-800 with respect to their heating temperature.

### 2.3. Preparation of Reed Panicle-Based Carbon Electrodes

The carbon sample (75 wt%), carbon black (20 wt%), and polytetrafluoroethylene (PTFE) binder (5 wt%) were mixed homogeneously to form a slurry and then pasted onto the nickel foam (1 × 1 cm^2^, current collector) with a mass loading of about 3.0 mg cm^−2^, followed by drying at 60 °C for 12 h to obtain the working electrodes.

### 2.4. Characterizations

The microstructures were measured by scanning/transmission electron microscope (SEM JEOL JSM-7500F, JEOL Ltd., Tokyo, Japan; TEM JEM-2800F, JEOL Ltd., Tokyo, Japan). Powder X-ray diffractometer (XRD) was used to characterize the crystal structures (2θ range of 5–90° with a scanning rate of 10°/min). X-ray photoelectron spectroscopy (XPS) measurement (Thermo Scientific K-Alpha, Waltham, MA, USA) was used to detect the surface chemical state of the obtained samples. Measurements of N_2_ adsorption–desorption at 77 K, employing the Brunauer–Emmett–Teller (BET) method to detect the specific surface area and the pore size distribution was estimated by density functional theory (DFT). Before analysis, the samples were degassed under vacuum (Vacuum degree: 3.071 × 10^−4^ Pa) at 200° for 4 h.

### 2.5. Electrochemical Measurements

#### 2.5.1. Three-Electrode System

In 6 M KOH solution, the electrochemical performance of the three-electrode system (platinum plate for the counter electrode and Hg/HgO electrode for the reference electrode) was investigated by cyclic voltammetry (CV), galvanostatic charge–discharge (GCD), and electrochemical impedance spectroscopy (EIS) on a CHI660E electrochemistry workstation (Chenhua, Shanghai, China) at room temperature. The CV curves of the three-electrode system were tested in the voltage range from −1 to 0 V at a scan rate of 2–100 mV s^−1^. GCD curves were tested at current densities ranging from 0.5 to 20 A g^−1^. EIS measurements were made in the frequency range of 0.01 Hz to 1 MHz.

The gravimetric specific capacitance (C) was computed by the following formula:

(1) $$C\;=\;\frac{I\Delta t}{m\Delta V}$$

where *I* (A) is the constant discharging current, Δ*t* (s) is discharge time, Δ*V* (V) is the potential region, and *m* (g) is the mass of active materials.

#### 2.5.2. Symmetric Supercapacitor

The electrochemical performance of the synthesized PC materials was also evaluated in a symmetrical device using a two-electrode system in 1 M Na_2_SO_4_ solution. All measurements were tested with an electrochemical workstation (CHI 660E).

By testing the two-electrode system, we can calculate the gravimetric specific capacitances via the following:

(2) $$C\;=\;\frac{4I\Delta t}{m_{t}V}$$

where *I* (A) refers to the discharge current, Δ*t* (s) is the discharge time, *m_t_* (g) is the total mass of the porous carbon material for two electrodes, and *V* (v) is the potential change.

Moreover, the specific energy (E, Wh ${\mathrm{kg}}^{-1}$) was calculated via the following equation:

(3) $$E\;=\;\frac{CV^{2}}{2\;\times \;3.6}$$

where *C* (F $g^{-1}$) represents the specific capacitance and *V* refers to the potential change.

The specific power (P, W ${\mathrm{kg}}^{-1}$) can also be obtained with the following formula:

(4) $$P\;=\;\frac{3600\;\times \;E}{\Delta t}$$

where *E* is the energy density and Δ*t* refers to the discharge time.

## 3. Results and Discussion

**Figure 1:**
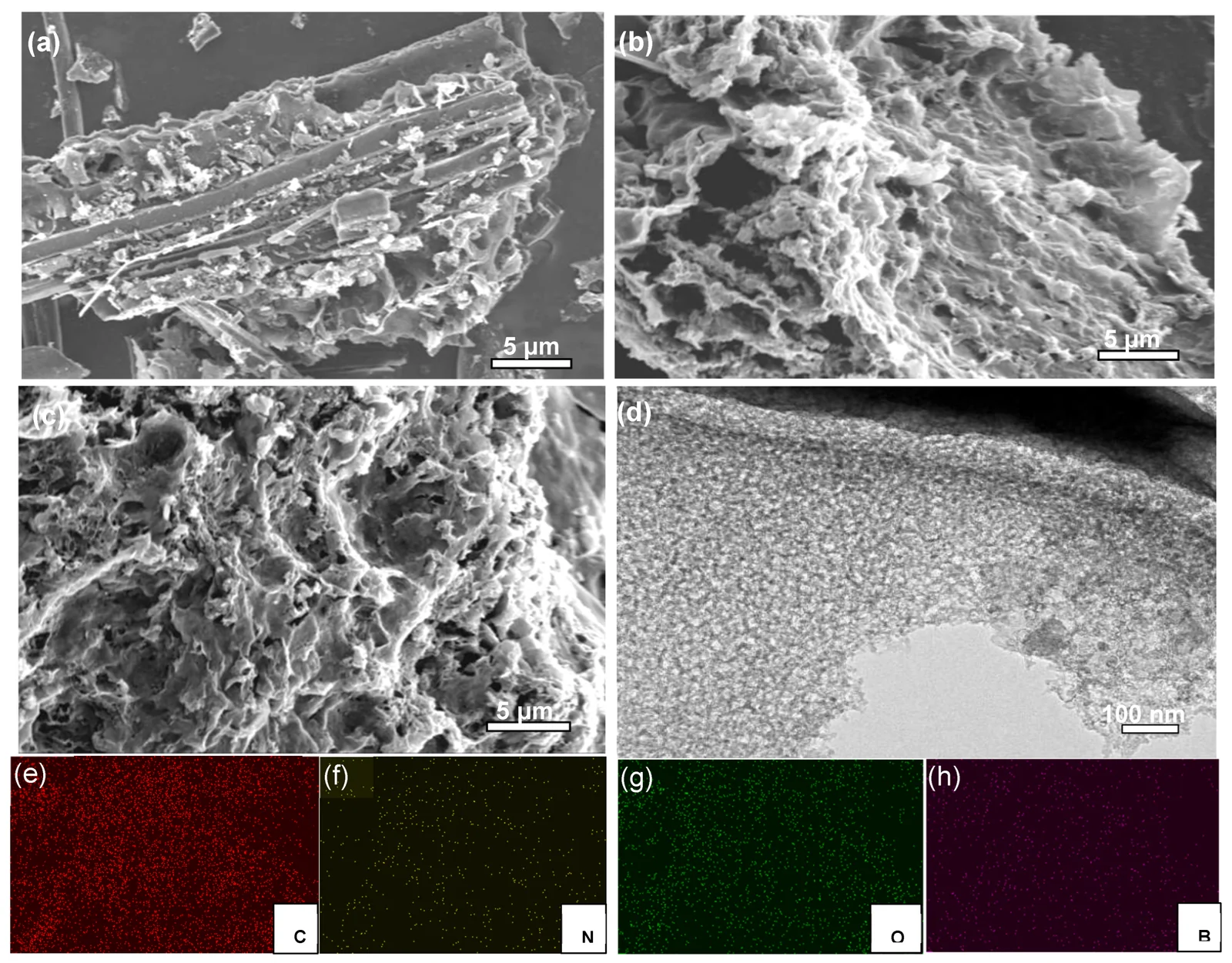
(**a**) SEM image of PC-600. (**b**) SEM image of PC-700. (**c**) SEM image of PC-800. (**d**) TEM image of PC-700. Elemental mapping images of PC-700: (**e**) C, (**f**) N, (**g**) O, (**h**) B.

The surface morphology and structure of the materials were characterized using scanning/transmission electron microscopy (SEM/TEM). As illustrated in Figure 1a–c, the SEM images of PC samples exhibit porous channels and interlaced lamellar architectures with a pervasive pore network, thereby affording plentiful active reaction sites and favoring mass transfer. Notably, as the temperature increases, the large pore structure becomes increasingly apparent, which is beneficial for shortening the distance of ion diffusion. From the corresponding element mapping image results (Figure 1e–h), carbon, nitrogen, oxygen, and boron were evenly spread-out throughout the entire carbon framework. Furthermore, the TEM image of the PC-700 samples demonstrates that there are numerous micropores and mesopores on the surface of the carbon skeleton. This unique hierarchical porous architecture provides short diffusion paths for ions and enhances interfacial charge transfer, thereby boosting the overall electrochemical kinetics. The formation of micropores and mesopores is attributed to the reaction between KHCO_3_ and carbon during high-temperature activation, which etches partial carbon and thereby generates abundant pores on the surface of the carbon material. The possible chemical reactions that may happen are shown in Equations (5)–(9) [32,33].

2KHCO_3_ → K_2_CO_3_ + CO_2_ + H_2_O (5)

K_2_O_3_ + C → K_2_O + 2CO (6)

K_2_CO_3_ → K_2_O + CO_2_ (7)

K_2_O + C → 2K + CO (8)

CO_2_ + C → 2CO (9)

**Figure 2:**
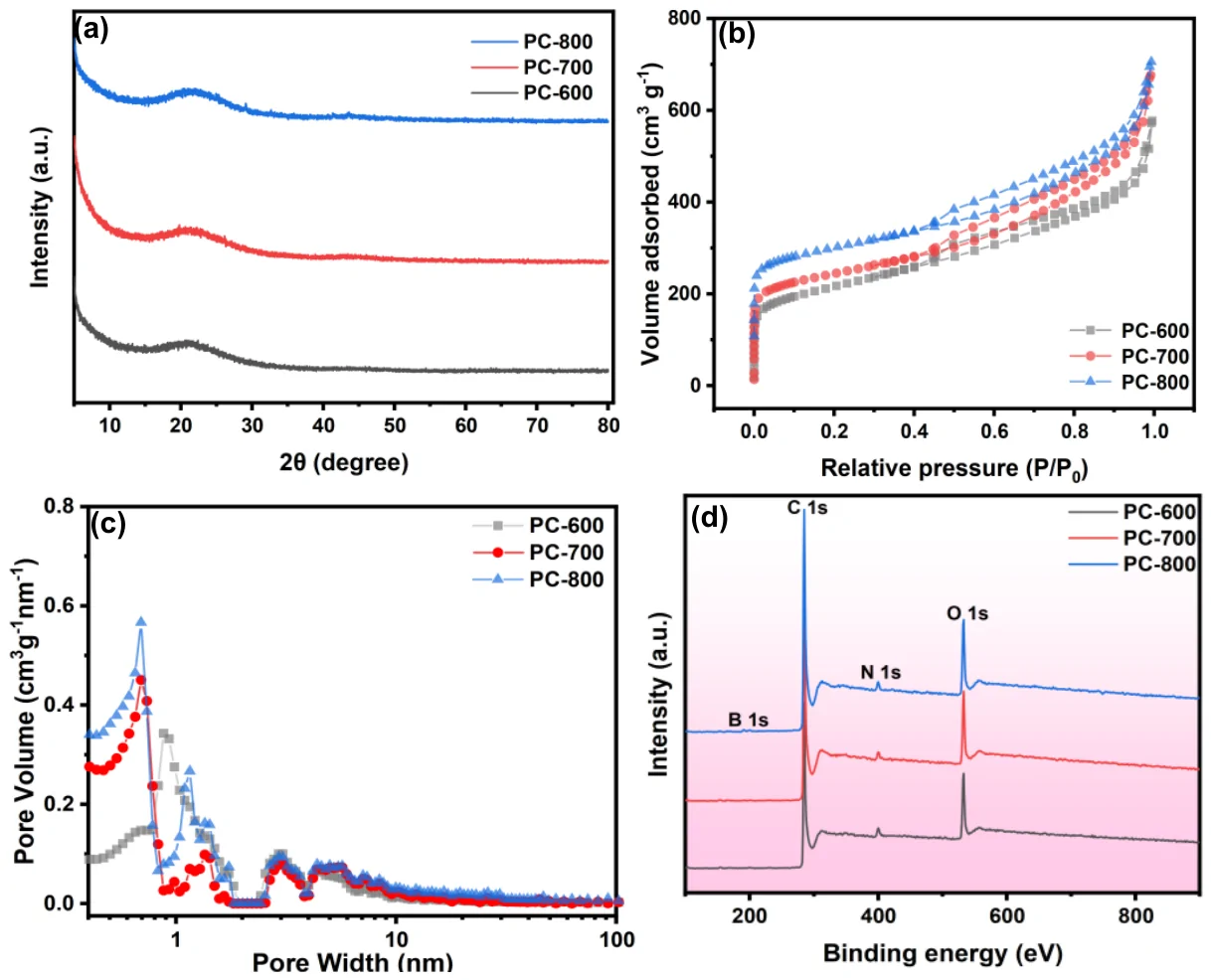
(**a**) XRD pattern; (**b**) N_2_ absorption–desorption isotherms; (**c**) pore size distribution curves of PC-600, PC-700, PC-800; and (**d**) XPS survey spectra of PC samples.

The crystal structure of the prepared porous carbon was tested by X-ray diffraction. As depicted in Figure 2a, all specimens exhibit a broad peak at 23.5° which is associated with the (002) crystallographic planes of disordered carbon [34,35]. Notably, a high peak intensity in the small-angle region indicates that the material has a porous structure and the disordered stacking of amorphous carbon tends to form numerous micropores and mesopores, thereby providing more ion adsorption sites.

N_2_ adsorption–desorption isotherms were measured to examine the pore structure of the materials. As shown in Figure 2b, all PC materials illustrate a Type IV isotherm with H3 hysteresis loops. At lower relative pressure (P/P_0_ < 0.05), all PC materials show a steep increase, signifying the presence of a significant amount of micropores. Furthermore, the H3-type hysteresis loop (0.5 < P/P_0_ < 0.9) confirms the lamellar structure of the carbon material, accompanied by the formation of abundant slit-shaped mesopores and macropores. The pore parameter of the samples is listed in Table 1. The specific surface areas of PC-600, PC-700, PC-800 are 758.64 m^2^ g^−1^, 882.77 m^2^ g^−1^, 977.76 m^2^ g^−1^, respectively. As shown in Figure 2c, when compared with PC-600, PC-700 and PC-800 contain more micropores, indicating that the KHCO_3_ etches the carbon matrix at elevated temperatures to generate micropores. Simultaneously, the PC-700 specimens possesses a massive micropore range from 0.8 nm to 2.0 nm and a rich mesopore range from 2.8 nm to 4.0 nm, as well as a range from 4.1 nm to 10 nm. The hierarchical porous architecture can not only provide massive active sites to form an interface double layer for energy storage but also provides ion fast channels [36,37].

**Figure 3:**
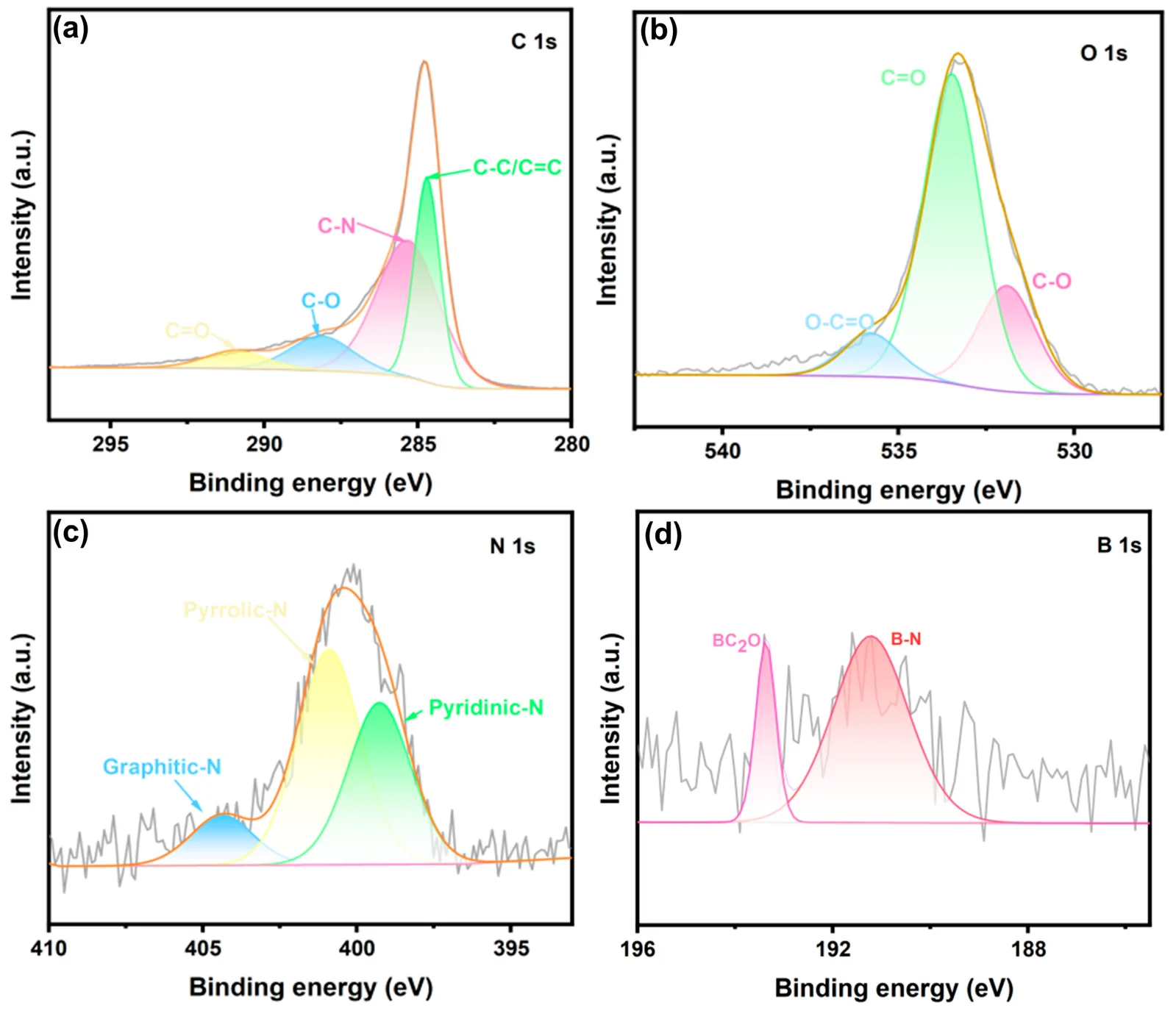
High resolution (**a**) C 1 s, (**b**) O 1 s, (**c**) N 1 s, (**d**) B 1 s.

The surface chemistry and status of the PC specimens were characterized via X-ray photoelectron spectroscopy (XPS). As seen in Figure 2d, 284.41 eV, 399.9 eV, 532.4 eV, and 191 eV correspond to C 1 s, N 1 s, O 1 s, and B 1 s. The atomic percentage of the four samples is shown in Table 2. The contents of N, O, B in PC-700 are 2.54 at%, 11.23 at%, and 2.59 at%, indicating the successful achievement of nitrogen and boron co-doping. As seen in Figure 3a, the C 1 s spectrum can be categorized into four peaks: 284.7 eV, 285.3 eV, 288.2 eV and 291.0 eV, corresponding to C-C/C=C, C-N, C-O and C=O [38], respectively. Apart from that, the O 1 s spectrum (Figure 3b) shows three peaks at 531.8 eV, 533.4 eV, 535.8 eV, representing C-O, C=O, O-C=O. Furthermore, as shown in Figure 3c, the high-resolution N1s spectrum of PC-700 is divided into three peaks, which are pyridinic-N (399.3 eV), pyrrolic-N (401 eV) and graphitic-N (404.3 eV) [39,40]. The B 1s spectrum (Figure 3d) shows two peaks at 191.2 eV and 193.4 eV, showing B-N and BC_2_O, respectively.

**Figure 4:**
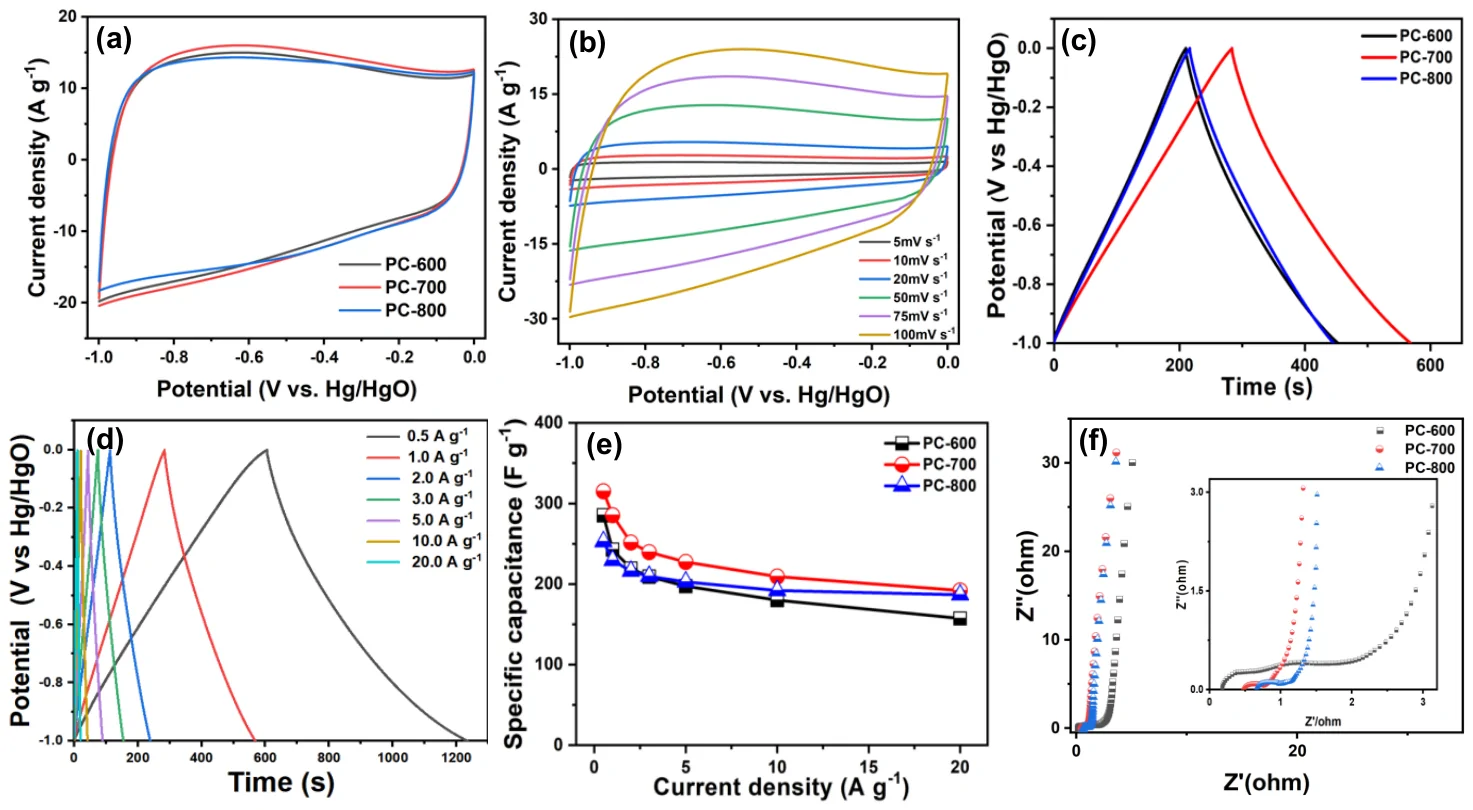
(**a**) CV curves of PC samples in different voltage ranges at 50 mV $s^{-1}$; (**b**) CV curves of PC-700 at various scan rate; (**c**) CP curves of PC samples at 1 A $g^{-1}$; (**d**) GCD curves of PC-700 at different current densities; (**e**) specific capacitance of PC samples; (**f**) Nyquist plot.

**Figure 5:**
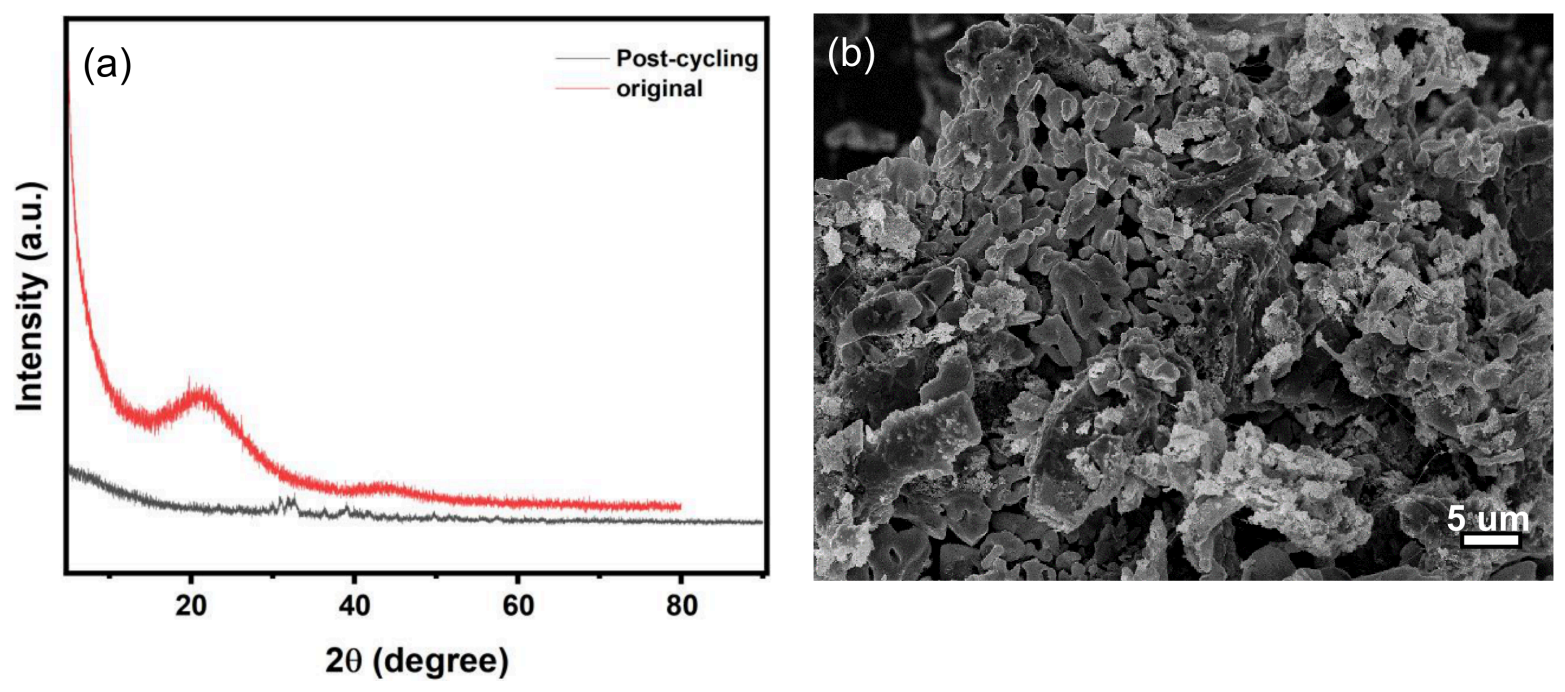
(**a**) The post-cycling XRD after 10,000 cycles tested at 200 mV $s^{-1}$. (**b**) The post-cycling SEM after 10,000 cycles tested at 200 mV $s^{-1}$.

A comprehensive electrochemical evaluation of the PC materials was conducted using a three-electrode system in a 6 M KOH electrolyte, employing cyclic voltammetry (CV), galvanostatic charge–discharge (GCD), and electrochemical impedance spectroscopy (EIS). As displayed in Figure S3 (Supplementary Materials), The reed panicle-derived carbon co-activated with KHCO_3_ and doped with ammonium borate exhibits the best electrochemical performance. As shown in Figure 4a, the CV curves of all samples exhibit superior quasi-rectangular configuration with a small redox peak, indicating that the capacity mainly comes from the electronics’ double layer capacitance and pseudocapacitance that are the result of the rich heteroatoms’ functional groups. Notably, the CV curve integrated area of PC-700 is larger than the others, meaning that the PC-700 has a higher specific capacitance. As depicted in Figure 4b, even at 100 mV s^−1^, it can still maintain a good rectangular shape, indicating superior rate behavior. As depicted in Figure 4c, the PC-700 electrode shows the longest discharge time, in alignment with the CV curves, suggesting the highest specific capacitance. As illustrated in Figure 4d, the specific capacitance was calculated according to the GCD curves. As illustrated in Figure 4e, the specific capacitance of PC-700 is 329.6 F g^−1^ at 0.5 A g^−1^, which is comparable to PC-600 (283.5 F g^−1^), PC-800 (253.2 F g^−1^) and other reported biomass-based porous carbons listed in Table 3 [41,42,43,44,45,46,47,48]. As illustrated in Figure 4f, the Nyquist plot of the PC-700 electrode in the low-frequency region possesses a steep linear curve, showing a good capacitive characteristic. PC-700 exhibits a lower charge-transfer resistance (R_ct_ = 0.16 Ω) than PC-600 (R_ct_ = 0.84 Ω) and PC-800 (R_ct_ = 0.24 Ω), indicating its most favorable charge-transfer kinetics and enhancing ion transport within the material, thereby contributing to superior electrochemical performance. This behavior can be primarily attributed to abundant micropores and interconnected pore channels within the materials. To assess whether electrochemical cycling alters the structure of samples, the PC-700 materials were evaluated after testing (post-cycling). As shown in Figure 5a, the post-cycling XRD shows a near vanishing of the broad peak at 23.5°, which may be attributed to the Turbostratic structure of the pristine sample, implying severe deterioration of the ordered carbon framework after prolonged cycling. Apart from that, the post-cycling SEM (Figure 5b) concurrently reveals marked particle agglomeration. The capacitive performance of the electrode relies mainly on short-range structure, surface active sites, and interlayer ion channels. However, the local coordination environment critical for charge storage may remain essentially intact. Meanwhile, post-cycling XRD and SEM were collected ex situ on relaxed electrodes, while the electrochemical data reflect dynamic operation and the observed structural changes and agglomeration could be localized and do not necessarily govern the overall capacitance. Moreover, the high carbon black content (20 wt%) builds a robust conductive network that effectively buffers any detrimental effect of agglomeration on electron transport. Hence, the structural evolution seen in XRD and SEM does not contradict the high capacitance retention.

**Figure 6:**
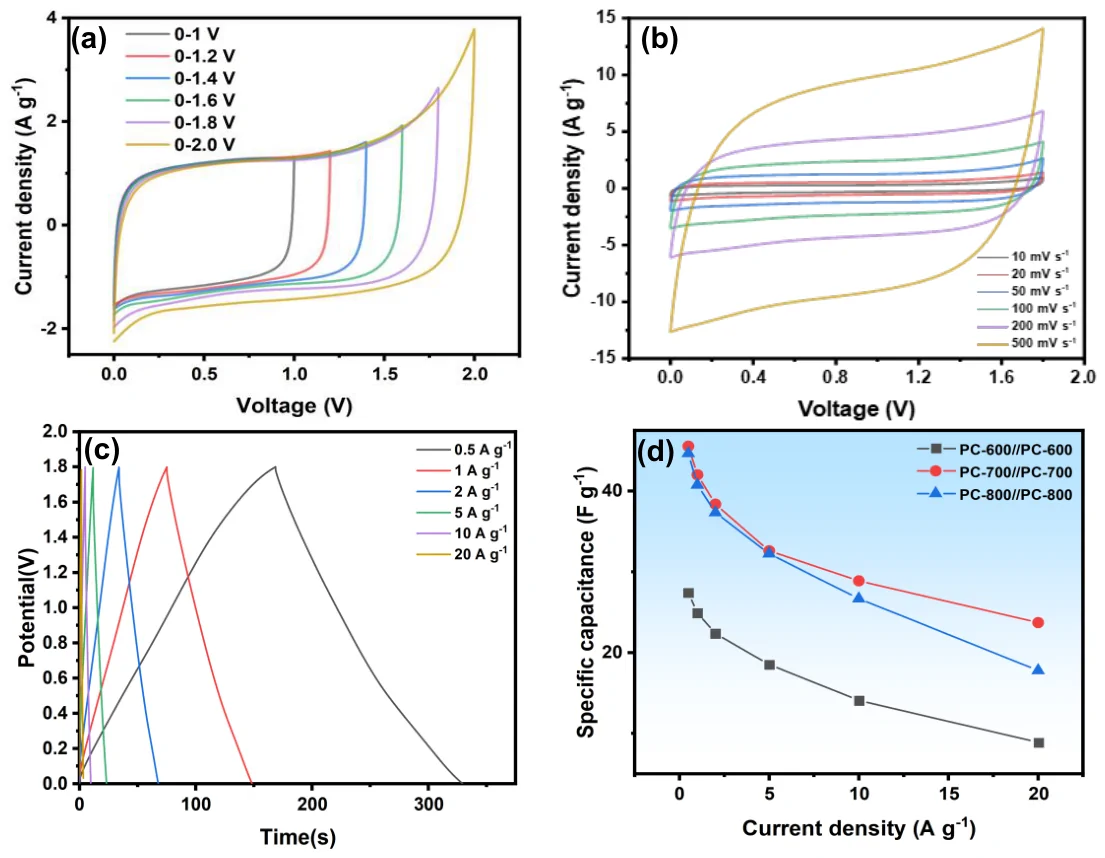
(**a**) CV curves of the PC-700//PC-700 symmetrical supercapacitor in different voltage ranges at 50 mV s^−1^; (**b**) CV curves of PC-700//PC-700 at various scan rates; (**c**) GCD curves of the PC-700//PC-700 symmetrical super capacitor at various current density; (**d**) the specific capacitances of the PC-600//PC-600, PC-700//PC-700, and PC-800//PC-800 symmetrical supercapacitors.

**Figure 7:**
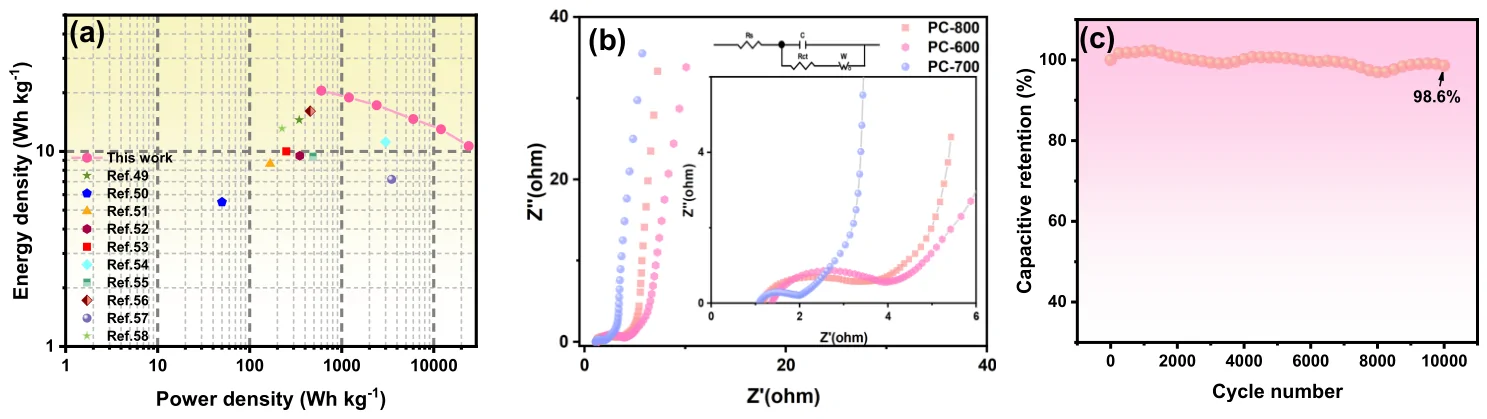
(**a**) Ragone plots of the PC-700//PC-700 symmetrical supercapacitor; (**b**) Nyquist plots; (**c**) electrochemical stability of the PC-700//PC-700 symmetrical supercapacitor for 10,000 cycles tested at 200 mV $s^{-1}$ [49,50,51,52,53,54,55,56,57,58].

To further validate the application potential of our materials, a symmetric supercapacitor was fabricated (Figure S1). As depicted in Figure 6a, the CV curve of the SSC in various voltage ranges at 50 mV s^−1^. When the voltage is increased to 2.0 V, the current of SSC displays a sharp rising trend, which is attributed to the decomposition of the electrolyte. Figure 6b presents the CV profile of SSC, suggesting negligible changes as the scan rate rises from 10 mV s^−1^ to 500 mV s^−1^, which means it exhibits ideal capacitive characteristics and high-rate performance. The GCD curves (Figure 6c) show a symmetric isosceles triangle without obvious voltage drop, indicating that the electrode material has an outstanding charge–discharge characteristic and rate capability. As shown in Figure S2 (Supplementary Materials), the PC-700 symmetric supercapacitor gives the largest integrated CV area. A clear reduction current appears below 0.6 V, suggesting that heteroatoms in the material contribute extra pseudocapacitance to the electrodes. The charge–discharge curves confirm this: Across all tested temperatures, the profiles are nearly linear and symmetric, with a noticeable bend below 0.6 V—matching the CV observations and further evidencing the pseudocapacitive role of heteroatom-containing functional groups. As depicted in Figure 6d, the PC-700 symmetric supercapacitor shows a specific capacitance of 45.6 F g^−1^ at 0.5 A g^−1^, which is higher than the 27.4 F g^−1^ at 0.5 A g^−1^ of PC-600//PC-600 and the 44.6 F g^−1^ at 0.5 A g^−1^ of PC-800//PC-800 and keeps 63.4% of its capacitance even at 10 A g^−1^. As illustrated in Figure 7a, due to the broad voltage range and large specific capacitance, the symmetric supercapacitor shows an energy density of 20.5 Wh kg^−1^ at a power density of 600 W kg^−1^, which is higher than those reported in other research [49,50,51,52,53,54,55,56,57,58]. The Nyquist curve of the PC-700//PC-700 supercapacitor was illustrated in Figure 7b. The curve exhibits a low semicircle in the high-frequency area associated with the interfacial layer resistance, while the semicircle in the medium-to-high-frequency region corresponds to the charge transfer resistance, and the 45° line in the low-frequency region represents the Warburg impedance. By fitting the Nyquist curve of the PC-700//PC-700 supercapacitor, its R_s_ and R_ct_ values are calculated to be 1.14 Ω and 0.59 Ω, respectively, which shows a relatively high conductivity. The comparison of resistance among PC-600//PC-600, PC-700//PC-700, and PC-800//PC-800 is listed in Table S1 (Supplementary Materials). The specific capacitance retention rate of the PC-700//PC-700 symmetric supercapacitor was maintained at 98.60% after 10,000 measurements at 200 mV s^−1^, indicating superior electrochemical behavior (Figure 7c).

## 4. Conclusions

To address the ecological disruption caused by the rampant overgrowth of reed, we herein present a sustainable and feasible strategy to upcycle this lignocellulosic biowaste into functional carbon materials via a facile one-step activation approach, materials which are subsequently employed as electrodes for symmetric supercapacitors. The prepared PC-700 materials possess high specific surface area and rich heteroatomic functional groups, which provide extra pseudocapacitance. PC-700 materials exhibit a more rational micro-/mesopore balance, wherein the mesopores function as ion-transport highways to ensure the accessibility of active sites embedded within the micropores. Therefore PC-700 materials are superior to PC-600 and PC-800 and show a specific capacitance of 329.6 F g^−1^ at 0.5 A g^−1^, a long lifespan, and excellent charge-transfer kinetics (R_ct_ = 0.16 Ω). Notably, the assembled PC-700 symmetric supercapacitor achieves an energy density of 20.5 Wh kg^−1^ at a power density of 600 W kg^−1^ and excellent electrochemical stabilization. Furthermore, post-cycling XRD and SEM were employed to further test the structural stability of the electrode material. Our findings establish reed panicles as a compelling biowaste-derived platform for high-efficiency carbon materials, offering a realistic pathway toward the commercialization of biomass-based supercapacitors.

## Figures and Tables

**Table 1:** Porosity and yield parameters of the PC carbon materials.

Samples	S_BET_ (m^2^ g^−1^) ^a^	S_mic_ (m^2^ g^−1^) ^b^	S_me_ (m^2^ g^−1^) ^c^	V_total_ (cm^3^ g^−1^) ^d^	D (nm) ^e^
PC-600	758.64	580.93	177.71	0.83	4.38
PC-700	882.77	777.85	104.92	1.03	4.67
PC-800	977.76	889.46	88.30	1.06	4.34

^a^ Specific surface area (S_BET_) was calculated with a modified Brunauer–Emmett–Teller (BET) method. ^b^ Micropore surface area (S_mic_) was obtained using the t-plot method. ^c^ Mesopore surface area (S_me_) was obtained using the t-plot method. ^d^ Total pore volume (V_total_) was estimated from the adsorbed amount at a relative pressure of 0.99. ^e^ Average pore diameter (D) was obtained from D = 4V_total_/S_BET_.

**Table 2:** The atomic percentages of the C, N, O and B of carbon materials from the XPS results.

Samples	C at%	N at%	O at%	B at%
PC-600	81.00	3.35	13.32	2.33
PC-700	83.64	2.54	11.23	2.59
PC-800	85.00	2.27	8.64	3.79

**Table 3:** Comparison of the specific capacitances of the reported biomass-derived carbon materials.

Biomass Source	Specific Capacitance (F g^−1^)	Electrolyte	References
Bamboo shoot shells	209 F g^−1^ at 0.5 A g^−1^	6 M KOH	[41]
Ginkgo leaves	186.5 F g^−1^ at 0.5 A g^−1^	4 M KOH	[42]
Ipomoea aquatica	287.2 F g^−1^ at 0.5 A g^−1^	6 M KOH	[43]
Rice husk	160.53 F g^−1^ at 0.5 A g^−1^	6 M KOH	[44]
Bio-oil derived from rice husk	248 F g^−1^ at 0.5 A g^−1^	6 M KOH	[45]
Cotton	103.9 F g^−1^ at 0.5 A g^−1^	6 M KOH	[46]
Cellulose aerogels	152.6 F g^−1^ at 0.5 A g^−1^	6 M KOH	[47]
Oak wood	174.4 F g^−1^ at 0.5 A g^−1^	6 M KOH	[48]
Reed spike	329.6 F g^−1^ at 0.5 A g^−1^	6 M KOH	This work

## Data Availability

The data that support the findings of this study are available from the corresponding author upon reasonable request.

## Supplementary Materials

The following supporting information can be downloaded at: https://www.mdpi.com/article/10.3390/nano16161035/s1, Figure S1: Assembly (a) and testing (b) of symmetrical capacitor; Figure S2: Electrochemical performance of electrodes fabricated at various carbonization temperatures: (a) CV curves at 20 mV s^−1^; (b) galvanostatic charge-discharge curves at 0.5 A g^−1^. The temperatures in the legends denote the carbonization temperatures, not the measurement temperatures; Figure S3: (a) CP curves of samples at 0.5 A g^−1^; (b) CV curves of samples in different voltage ranges at 50 mV s^−1^; (c) specific capacitance of PC samples; Table S1: Comparison of resistances among the PC-600//PC-600, PC-700//PC-700, and PC-800//PC-800 supercapacitors.
