# Supplementary material for: Conversion of Biological Waste into Porous Carbon with Hierarchical Porous Architecture for High-Performance Supercapacitors
Source: Nanomaterials (Basel). 2026 Aug 20;16(16):1035. doi: 10.3390/nano16161035 (PMC13516699; doi:10.3390/nano16161035)
Supplement: Supplementary file 1 [file nanomaterials-16-01035-s001.zip › nanomaterials-4410957-supplementary.pdf]

Supplementary materials

Conversion of Biological Waste into Porous Carbon with Hierarchical Porous Architecture for High-Performance Supercapacitors

Yueyang Lu [1], Siyu Han [2], Yizhe Wang [1], Zekun Tang [1] and Xiaoliang Wu [1,*]

[1] State Key Laboratory of Utilization of Woody Oil Resource, College of Chemistry, Chemical and Resource Utilization, Northeast Forestry University, Harbin 150000, China; 15736877356@163.com (Y.L.); wangyizhe05@vip.163.com (Y.W.); tangzekun2004@163.com (Z.T.)

[2] Jiamusi Ecological Environment Technology Center, No. 676 Chang'an West Road, 150040 Jiamusi City, China; H13394543421@163.com(S.H.)

* Correspondence: wuxiaoliang90@163.com

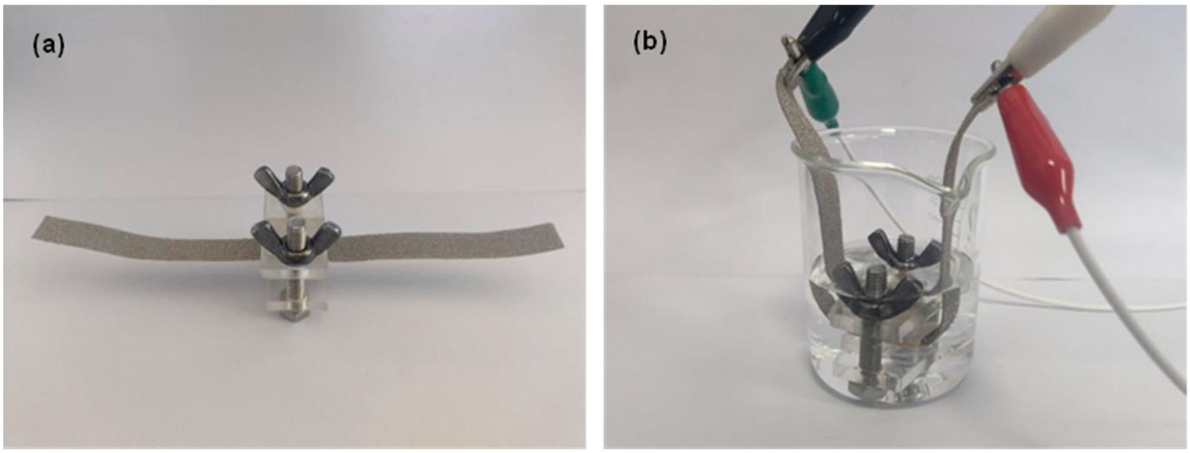

Figure S1. Assembly (a) and testing (b) of symmetrical capacitor.

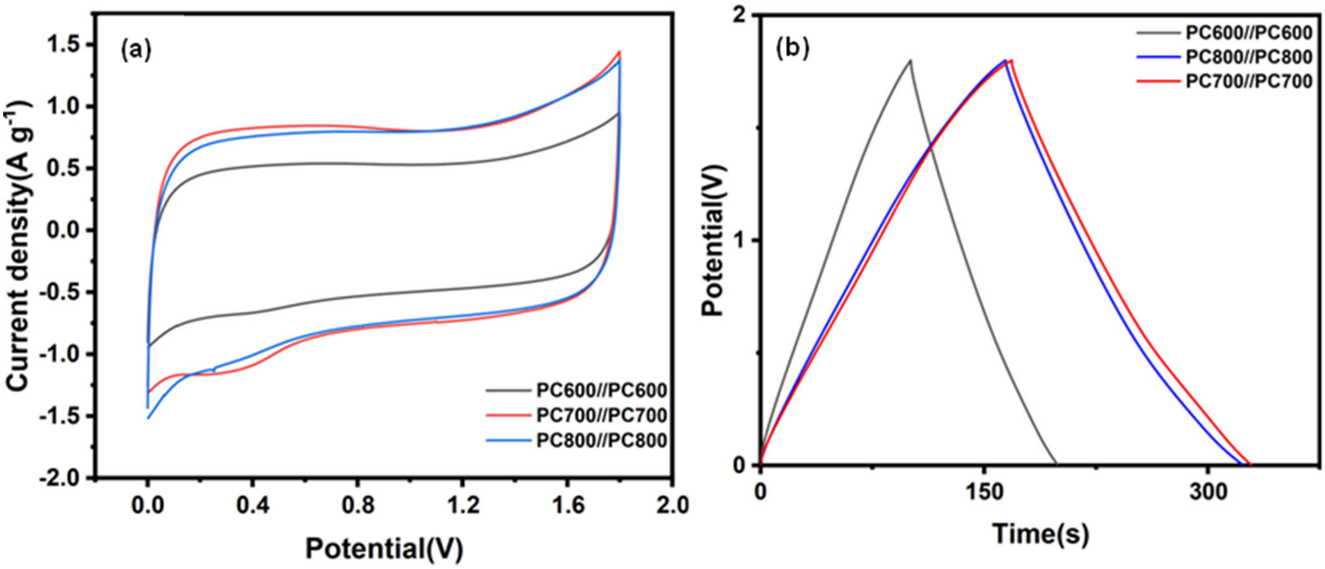

Figure S2. Electrochemical performance of electrodes fabricated at various carbonization temperatures: (a) CV curves at 20 mV s^{-1}; (b) galvanostatic charge - discharge curves at 0.5 A g^{-1}. The temperatures in the legends denote the carbonization temperatures, not the measurement temperatures.

Table S1. Comparison of resistances among the PC-600//PC-600, PC-700//PC-700, and PC-800//PC-800 supercapacitors.

Samples	R_s	R_{ct}
PC-600	1.49	1.826
PC-700	1.14	0.59
PC-800	1.27	1.61

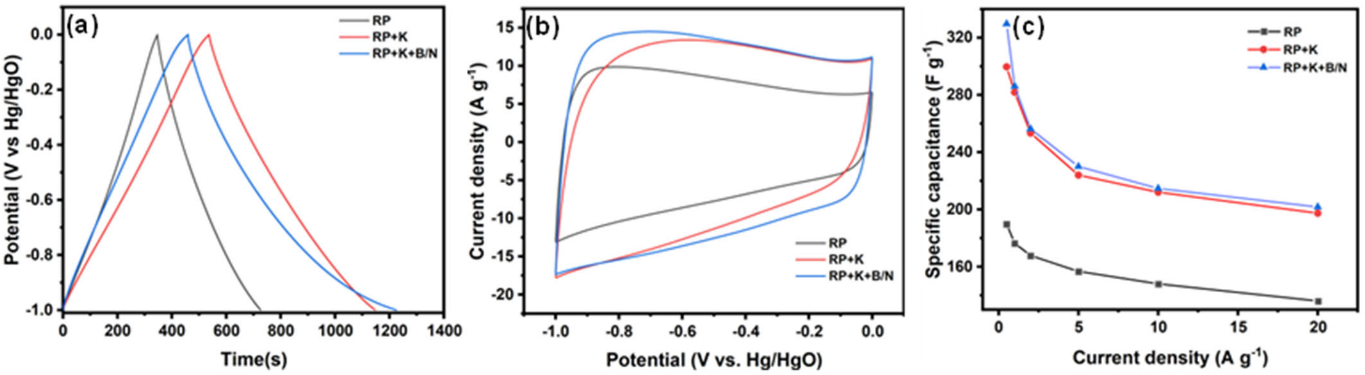

Figure S3. (a) CP curves of samples at 0.5 A g^{-1}; (b) CV curves of samples in different voltage ranges at 50 mV s^{-1}; (c) specific capacitance of PC samples.
